# Modified Mineral Trioxide Aggregate—A Versatile Dental Material: An Insight on Applications and Newer Advancements

**DOI:** 10.3389/fbioe.2022.941826

**Published:** 2022-08-09

**Authors:** C. Pushpalatha, Vismaya Dhareshwar, S. V. Sowmya, Dominic Augustine, Thilla Sekar Vinothkumar, Apathsakayan Renugalakshmi, Amal Shaiban, Ateet Kakti, Shilpa H. Bhandi, Alok Dubey, Amulya V. Rai, Shankargouda Patil

**Affiliations:** ^1^ Department of Pedodontics and Preventive Dentistry, Faculty of Dental Sciences, M.S. Ramaiah University of Applied Sciences, Bangalore, India; ^2^ Department of Oral Pathology and Microbiology, Faculty of Dental Sciences, M.S. Ramaiah University of Applied Sciences, Bangalore, India; ^3^ Department of Restorative Dental Science, Division of Operative Dentistry, College of Dentistry, Jazan University, Jazan, Saudi Arabia; ^4^ Department of Conservative Dentistry and Endodontics, Saveetha Dental College, Saveetha Institute of Medical and Technical Sciences, Chennai, India; ^5^ Department of Preventive Dental Sciences, Division of Pedodontics, College of Dentistry, Jazan University, Jazan, Saudi Arabia; ^6^ Department of Endodontics, College of Dentistry, King Khalid University, Abha, Saudi Arabia; ^7^ Department of Pediatric Dentistry, Preventive Division, Riyadh Elm University, Riyadh, Saudi Arabia; ^8^ Department of Restorative Dental Science, Division of Operative Dentistry, College of Dentistry, Jazan University, Jazan, Saudi Arabia; ^9^ Department of Cariology, Saveetha Dental College and Hospitals, Saveetha Institute of Medical and Technical Sciences, Saveetha University, Chennai, India; ^10^ Department of Preventive Dental Sciences, Division of Pedodontics, College of Dentistry, Jazan University, Jazan, Saudi Arabia; ^11^ Department of Maxillofacial Surgery and Diagnostic Sciences, Division of Oral Pathology, College of Dentistry, Jazan University, Jazan, Saudi Arabia; ^12^ Centre of Molecular Medicine and Diagnostics (COMManD), Saveetha Dental College and Hospitals, Saveetha Institute of Medical and Technical Sciences, Saveetha University, Chennai, India

**Keywords:** Mineral Trioxide Aggregate, MTA, Modified MTA, Drug Delivery, Dicoloration

## Abstract

Mineral Trioxide Aggregate (MTA) has been a material of revolution in the field of dentistry since its introduction in the 1990s. It is being extensively used for perforation repairs, apexification, root-end filling, obturation, tooth fracture repair, regenerative procedures, apexogenesis, pulpotomies, and as a pulp-capping material because of its desired features such as biocompatibility, bioactivity, hydrophilicity, sealing ability, and low solubility. Even though its application is wide, it has its own drawbacks that prevent it from reaching its full potential as a comprehensive replacement material, including a long setting time, discoloration, mud-like consistency, and poor handling characteristics. MTA is a material of research interest currently, and many ongoing studies are still in process. In this review, the newer advancements of this versatile material by modification of its physical, chemical, and biological properties, such as change in its setting time, addressing the discoloration issue, inclusion of antimicrobial property, improved strength, regenerative ability, and biocompatibility will be discussed. Hence, it is important to have knowledge of the traditional and newer advancements of MTA to fulfill the shortcomings associated with the material.

## Introduction

The practice of endodontic treatment has changed significantly over the past 200 years, with considerable advancements in technology and techniques. The conventional approach has undergone various changes, owing to rising patient demand for tooth preservation and improvements in material science and novel technology. Mineral Trioxide Aggregate (MTA) is a potent material in endodontics that has changed the prognosis of patients with the worst clinical condition of their teeth (Kaur et al., 2017). Despite the fact that the oral environment is typically damp, all dental products perform best in a dry environment. MTA is a Product developed using Portland cement and bismuth oxide (Camilleri, 2015).

**FIGURE 1:**
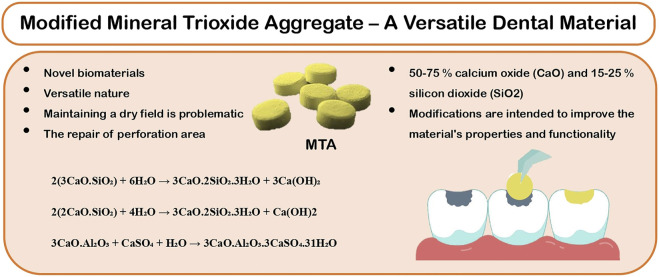
Properties that make modified MTA a versatile dental material.

The MTA was designed for certain clinical applications where maintaining a dry field is problematic, such as retrograde endodontic filling and the repair of perforated areas. Extended usage includes apexification, dressing over pulpotomies (PP), as a pulp-capping (PC) material, and sealer cement. The versatile nature of the MTA’s application demanded the necessity for new preparations that included additives with cement, which is an original or combination of radiopacifiers. These modifications are intended to improve the material’s properties and functionality (Figure1).

**FIGURE 2:**
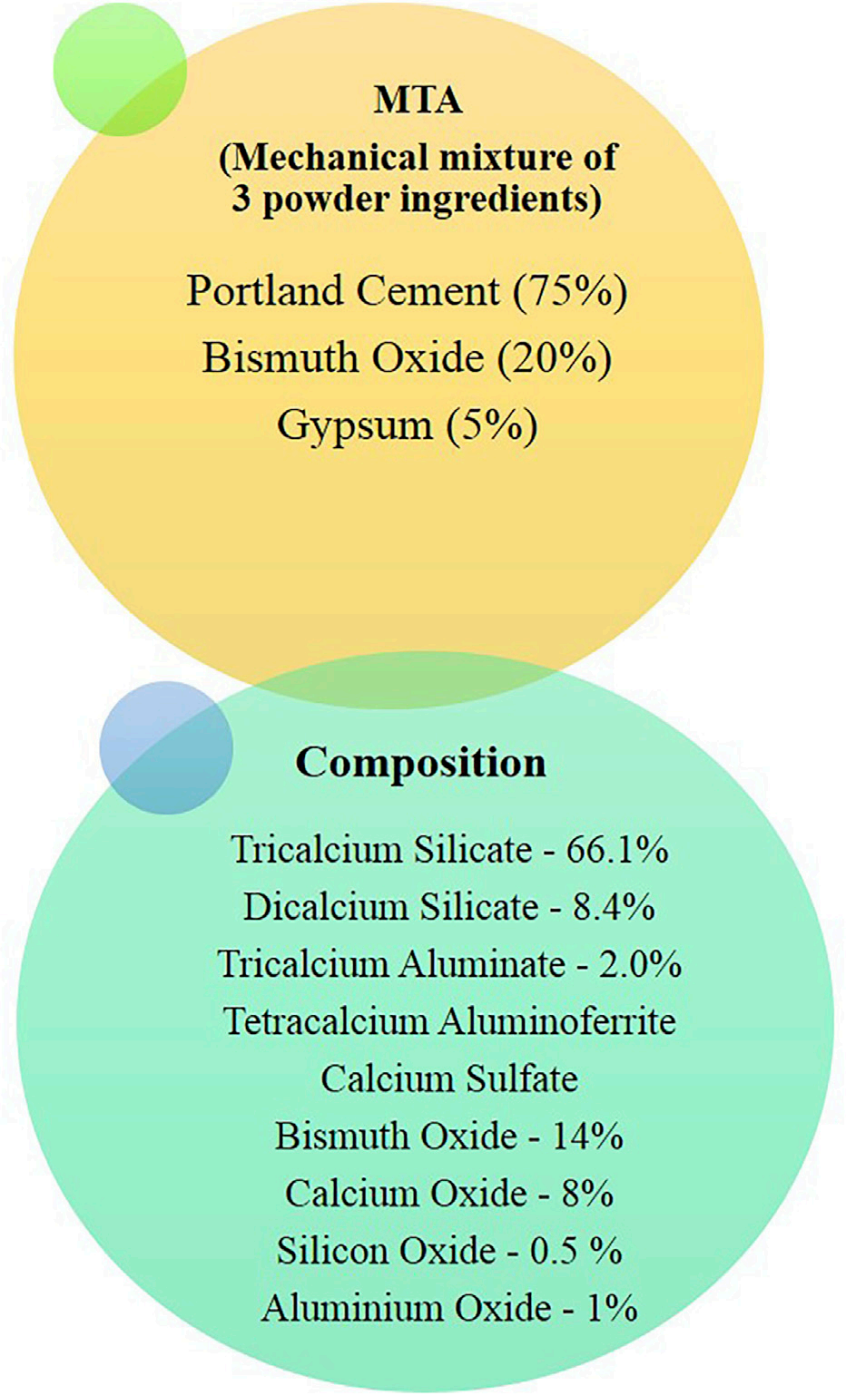
Composition of modified MTA.

MTA is composed of Portland cement with bismuth oxide and gypsum. Calcium, silicon, and aluminum are the other constituents of cement. Tricalcium and dicalcium silicates and tricalcium aluminate are the primary component (Figure2). MTA is made of 50–75% calcium oxide (CaO) and 15–25% silicon dioxide (SiO_2_) by weight. They make up 70–95% of cement (Camilleri, 2015). When the aforementioned raw ingredients are combined, then tricalcium silicate, dicalcium silicate, tricalcium aluminate, and tetracalcium aluminoferrite are formed. MTA is Type 1 Portland cement (American Society for Testing Materials), with a fineness (Blaine number) ranging from 4,500 to 4,600 cm^2^/g. For easier dental radiographic diagnosis, bismuth oxide is added to the cement (Camilleri, 2008).

X-ray diffraction analysis is used to determine the phases formed in MTA. Tricalcium silicate, dicalcium silicate, and bismuth oxide exhibited peaks in unhydrated MTA. Other phases, such as dicalcium silicate and tricalcium aluminate, are present in trace amounts (Camilleri, 2015). The original Portland cement formulation was replaced with tricalcium silicate to avoid the inclusion of an aluminum phase and to eliminate trace elements. Bismuth oxide was replaced with alternative radiopacifiers. Because of its many functions, MTA can come into contact with a range of oral environmental factors, including blood, saliva, tissue fluids, dental restorative materials, tooth structure, and even air. The quality and setting of the material are affected under these conditions (Tawil et al., 2015).

The setting time, mechanical properties, discoloration, manipulation, and mud-like consistency of cements have been a major technical concern of traditional MTA for a long time. More recently, these practical issues have emerged as a key source of concern for applications in the field of dentistry, particularly when it comes to the setting time, which should be maintained to a minimum. The goal of this review is to use an evidence-based dental approach to systematically evaluate the literature of modified MTA in order to overcome these shortcomings when compared to commercially available MTA.

## History

Portland cement was first used in dental literature in 1878 by Dr. Witte in Germany, who published a case report on utilizing the material to plug root canals. Joseph Aspdin, an Englishman, invented Portland cement in 1824. Over a century later, Dr. Mahmoud Torabinejad and his co-inventor, Dean White, received two US patents for MTA.

MTA was originally published in the dental literature in 1993 and was approved by the FDA in 1998. In 1998, Dentsply, Tulsa Dental, Johnson City, TN, United States, commercialized the original MTA as ProRoot MTA. The “Tooth-colored ProRoot MTA” was first introduced in 2002 and was later patented. ProRoot MTA gray and white versions had comparable compositions, while the tooth-colored versions use white Portland cement, which has less iron content.

Following the ProRoot, Dentsply introduced more MTA formulations. MTA Angelus (Angelus, Londrina, Brazil/Clinician’s Choice, New Milford, CT) was first introduced in Brazil in 2001 and acquired FDA approval in 2011, allowing it to be sold in the United States (Tawil et al., 2015). Angelus was the first to introduce it, and it included both gray and white formulations. Bismuth oxide with Portland cement was used in MTA Angelus. It contained different amounts of tricalcium and dicalcium silicate than ProRoot MTA and other cements (Camilleri, 2008). According to the manufacturer, the absence of gypsum decreased the material’s setting time. Hence, MTA Angelus was found to set in less than 50 min, compared to that of ProRoot MTA, which has been claimed to take over 2 h to set (Camilleri, 2015). When compared to ProRoot MTA, MTA Angelus had a lower level of bismuth oxide, which explains the reduced radiopacity of the material. The first MTA products were gray, and this formulation was the focus of much of the early research. The white form of MTA was introduced to the market in 2002 in response to staining problems raised when MTA residues were left in the clinical crown (Tawil et al., 2015).

Next, MTA Plus was introduced by Avalon Biomed, a firm based in the United States. The specific surface area of MTA Plus is 1.537 m^2^/g, which is larger than the other MTAs’ values. Because the specific surface area is greater, more surface is available for the cement reaction, resulting in a faster reaction rate. Using the Brunauer–Emmett–Teller (BET) gas adsorption method, ProRoot MTA and MTA Angelus were shown to have a similar fineness of 1 m^2^/g (Camilleri, 2015).

The categorization of calcium silicate (CS)-based materials has been proposed based on chemistry (Zafar et al., 2020) (Table 1). Despite numerous studies on the various materials available for the procedure, no material has yet been discovered that meets all of the criteria for an ideal material, such as biocompatibility, apical seal, and microleakage, and research is still ongoing.

**TABLE 1:** Classification of MTA as bioactive materials (Zafar et al., 2020).

Generation	Bio-active materials
First-generation	Gray MTA
	White MTA
Second-generation	Modifications to MTA
	MTA Angelus
Third-generation	Endo CPM (cement/Portland modified)
	iRootSP (also retailed as EndoSequence BC and SmartPaste Bio)
	MTA Obtura Tech Biosealer Endo
	• New endodontic cement/calcium-enriched mixture
	• Bioaggregate
	• Biodentine
	• Ortho MTA
	• MTA Plus
	• Generex A and Generex B
Fourth-generation	Hybrid cements
	• Calcium phosphate/calcium silicate/bismutite cement
	• NRC (incorporating HEMA (2- hydroxyethyl methacrylate))
	• MTA with 4-META/MMA- TBB (4-methacryloxyethyl trimellitateanhydrate in methyl methacrylate initiated by tri-n- butyl borane)
	• Light-cured cements including TheraCal LC

### Setting Reaction of MTA

MTA, being hydrophilic, requires moisture to set. Therefore, it is unhindered by blood or water, as moisture is required for a better setting of the material. The required hydration for setting is provided by a moist cotton pellet placed temporarily (until the next appointment) in indirect contact and/or on the surrounding tissues. The hydration reaction during setting occurs between tricalcium silicate and dicalcium silicate to form calcium hydroxide and calcium silicate hydrate gel, producing an alkaline pH. The released calcium ions diffuse through the dentinal tubules and increase their concentration over time as the material sets. The setting reaction of MTA is shown in the following reaction (Camilleri, 2015).

2(3CaO.SiO₂) + 6H₂O → 3CaO.2SiO_2_.3H_2_O + 3Ca(OH)_2_,
2(2CaO.SiO_₂_) + 4H_₂_O → 3CaO.2SiO₂.3HO + Ca(OH)_2_,
3CaO.Al₂O₃ + CaSO₄ + H₂O → 3CaO.Al₂O₃.3CaSO₄.31H₂O.

## Challenges Attributed to MTA Usage in Clinical Settings

Evidence-based practice is essential for dental practitioners in the new millennium. MTA is a biomaterial that has a significant impact in the dental field. Though MTA has a wide range of applications, it has limitations like discoloration, mud-like consistency, long setting time, leakage, solubility, and difficulty in manipulation that hinder it from achieving its full potential. Evidence-based practitioners who desire to adopt the most recent and best available evidence into their practices are sometimes perplexed by MTA research’s exponential expansion since 1993, which has resulted in the existence of various modified materials and multiple randomized clinical trials with inhomogeneous findings.

**FIGURE 3:**
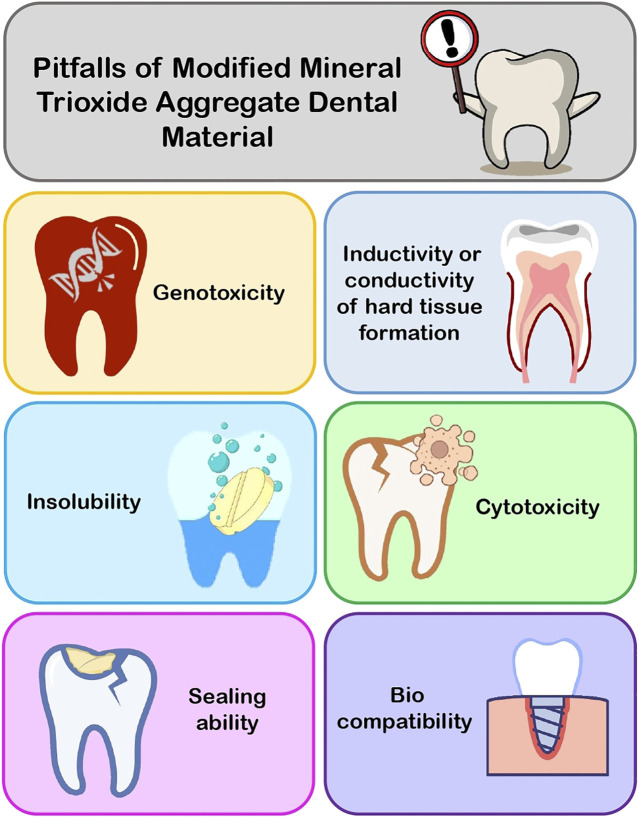
Pitfalls of modified MTA.

Modifications to MTA and items with different compositions but comparable applications are addressed here in order to assist dentists in selecting the proper material for fixing difficult instances with maximum competence. Many researchers suggested that changes to MTA and the introduction of new biomaterials for use in perforation repair, root-end filling, pulp capping, and other procedures necessitated testing for biocompatibility, cytotoxicity, genotoxicity, sealing ability, inductivity or conductivity of hard tissue formation, and insolubility before recommending them for clinical use. In the following text, the shortcomings of MTA and its modified aspects are discussed (Figure3).

### Discoloration

Tooth discoloration has been linked to bismuth oxide and calcium silicate (Parirokh and Torabinejad, 2010a; Krastl et al., 2013). The usage of both gray MTA (GMTA) and white MTA (WMTA) has also been linked to tooth discoloration. Reports indicate that gray MTA, white MTA Angelus, and white ProRoot MTA have strong staining potential, whereas Retro MTA, MM-MTA, and MTA Ledermix have least staining potential (Możyńska et al., 2017). Some clinical cases report dark discolorations of the teeth and gums after the MTA use (Karabucak et al., 2005; Berger et al., 2014; D’mello and Moloney, 2017). Tooth discoloration was also observed after using MTA in primary molars for pulpotomy. Nearly 60% of the cases have been reported with tooth discoloration (Naik and Hegde, 2005; Belobrov and Parashos, 2011).

GMTA caused clinically noticeable crown discoloration after 1 month, whereas the total color change caused by WMTA exceeded the perceptible threshold for the human eye after 3 months, implying that GMTA should be avoided in the esthetic zone and WMTA should be used with caution when filling pulp chambers with the materials (Camilleri, 2015). WMTA samples showed discoloration 3 days after inserting the material into a mold that was in contact with phosphate buffer solution (PBS). The reason for discoloration is debatable, linked mainly to interaction between bismuth oxide and the collagen of the tooth tissue and sodium hypochlorite, which is usually used in root canal therapy. When Boutsioukis et al. studied the removal efficiency of MTA as a root canal filler material in 2008, they detected deep root discoloration in the majority of the specimens filled with Angelus gray MTA (AWMTA). Arsenic present in many Portland cements and MTA can present discoloration. Iron and manganese have also been proposed as possible causes of discoloration (Boutsioukis et al., 2008). In a 2005 study on arsenic release from MTA, Duarte et al. found that the amount of arsenic emitted from MTA is extremely low. The presence of ferric oxides in MTA and their stabilising effect on arsenic in this material, as well as MTA's insolubility and use of modest doses of MTA for therapeutic purposes, may limit arsenic release into tissue fluids, which could cause toxicity (Duarte et al., 2005). However, the solubility of some Portland cements and the release of arsenic from these materials have raised concerns. A comparative study was carried out to evaluate the tooth discoloration using calcium-enriched mixture cement, Portland cement, and MTA mixed with propylene glycol (MTA-PG) in comparison to WMTA. The results showed that after 6 months, Portland cement had the most discoloration and MTA and MTA-PG had the least discoloration effect (Salem-Milani et al., 2017).

### Difficulty in Manipulation of MTA

MTA is a difficult cement to handle due to its granular consistency (Kogan et al., 2006), reduced setting time (Camilleri et al., 2005), and initial looseness of the material (Fridland and Rosado, 2003; Kogan et al., 2006; Coomaraswamy et al., 2007). When the mixture begins to dry, it loses its cohesiveness and becomes very difficult to handle the cement (Lee, 2000). The consistency of the freshly mixed material, which is commonly described as gritty or sandy, is another issue with the original MTA formulation. The original formulation is hand spatulated, making distribution to the surgical site challenging. To make handling easier, Teflon sleeves and pluggers particularly intended for placing MTA, mainly developed carriers for dispersing MTA, and scooping MTA out of grooves in a plastic block have all been employed. In one study, the hand and ultrasonic installation of varying thicknesses of MTA in high density polyethylene (HDPE) tubes were compared. Radiographic and microscopic analyses revealed that the manual method produced superior adaptation with fewer voids than the ultrasonic method for all thicknesses (Parirokh and Torabinejad, 2010b).

### Complexity as an Obturating Material

MTA could provide significant benefits like superior physiochemical and bioactive properties, when used as a root canal obturation material. MTA is an effective obturating material for retreatment; obturation is combined with root-end resection, apexification, internal resorption, dens in dente, and in conventional endodontic therapy. Obturating with MTA enhances the prognosis and retention of the natural teeth in both conventional and complex therapies.

Although MTA presents significant advantages, it has some disadvantages when used as obturating material. GMTA can discolor teeth if it is placed in the coronal part or near the cementoenamel junction (CEJ) in anterior teeth. This is due to the reduction of ferrous ions (FeO) in the dentinal tubules, which may increase over time (Asgary et al., 2005). It could be a significant issue in anterior aesthetics without PFM restorations and if ceramic crowns and veneers are not properly opaqued in the laboratory. Obturation of MTA in case of curved canals after placement is challenging for curing and removal of the material. Hence, MTA obturation is considered as a permanent filling material and therefore treated in the possible event of failure by surgical resection of the root end. Another relatively insignificant disadvantage is ProRoot MTA that has slow setting time and might take 2.5–4.0 h to set and 21 days to cure completely. Restoration using MTA in the curved canal should be considered a permanent filling for root canal obturation. The difficulty in obturating curved root canals, the potential for discoloration, and the extended setting time are all disadvantages of utilizing MTA as a root canal filling material (Torabinejad et al., 1995; Boutsioukis et al., 2008; Mathew et al., 2021).

### Long Setting Time

GMTA’s initial and final setting times are much longer than that of WMTA. The lower amounts of sulfur and tricalcium aluminate in WMTA account for the longer setting time when compared to Portland cement (Parirokh and Torabinejad, 2010b). One of the reasons why MTA should not be implemented in a single visit is its extended setting time. In comparison to other retrograde filling materials such as amalgam, Super-EBA, and Intermediate restorative material, MTA has a longer setting time (2 h and 45 min). MTA is unsuitable for most clinical applications due to its extended setting time. Different powder-to-water ratios are common in clinical practice because the quantity given is rarely used in a single application and have an impact on MTA’s characteristics, resulting in a prolonged setting time (Camilleri, 2015). This has been mentioned as one of the material’s flaws. According to researchers, WMTA has a much shorter setting time than GMTA. When samples are maintained under dry conditions, they affect MTA setting time and bacterial leakage. MTA should not be placed in one visit without external moisture since two-sided hydration results in greater flexural strength than one-sided hydration (Parirokh and Torabinejad, 2010b).

### Washout Effect

One of MTA’s disadvantages is washout or the tendency of freshly made cement paste to breakdown when it comes into contact with blood or other fluids. When cleaning an osteotomy site, a root-end filling material can washout, resulting in a weakened root-end seal and its consequences. It has been demonstrated that adding carboxymethyl chitosan or gelatin to cement based on calcium silicates (the primary components of Portland cement in MTA) improves washout resistance (Kim et al., 2011; Formosa et al., 2013).

### Solubility

MTA’s solubility is a point of contention among researchers. MTA solubility is found to be minimal or non-existent in most investigations. However, a long-term study, which found enhanced solubility, suggests that the powder-to-water ratio may alter the degree of solubility. MTA porosity and solubility increase with larger water-to-powder ratios. More water would boost calcium release from MTA, according to the authors. Another factor contributing to MTA’s insolubility is the presence of bismuth oxide, which is insoluble in water (Parirokh and Torabinejad, 2010b).

### Affordability of the Material

MTA is a dental material that has a variety of endodontic uses. According to a study, MTA is commonly used in postgraduate endodontic training programs in the United Kingdom, and its expensive cost is seen as the main barrier to its utilization. In the United Kingdom study, nearly half of the respondents said they wanted more information on how to utilize MTA in clinical practice. In case of direct pulp capping MTA is found to be more cost effective than calcium hydroxide. According to consultant pediatric dentist surveys, the majority of pediatric dentists do not use MTA due to its high cost (Ha et al., 2016).

## MTA Modifications With Improved Characteristics and Applications

Despite the fact that MTA is a hydraulic material, it is never exposed to water in clinical dentistry. The substance has been recommended for usage as a filling material for root ends, as well as for repair in perforation areas, apexification, pulpotomy treatments, and for pulp capping. MTA has recently been introduced as a sealant for root canals. The generation of by products, that is, calcium hydroxide and the hydraulic nature of MTA, is responsible for all of its beneficial properties (Camilleri, 2015). MTA interacts with a variety of environments depending on the material application. When MTA is employed for repair of the perforation area, as a material for root-end filling, and to a lesser extent, in the case of direct pulp capping (DPC), blood comes into contact with it. Blood obstructs the hydration of the material and lowers MTA microhardness (Camilleri, 2015). Material characteristics and failure to set are also influenced by tissue fluids, including serum.

The mixing and administration technique is the key problem in the clinical setting with initial MTA preparation (Camilleri, 2015). MTA is traditionally blended by manipulating the powder on a mixing pad by combining the solid and liquid components. Alternative techniques for mixing, such as the use of an amalgamator, have also been studied. There is a widely held idea among researchers about powder and cement that is the surface area and initial setting time are inversely proportional. In other words, as the surface area of the powder increases, the likelihood of the particles reacting with water increases, resulting in a faster hydration process and a shorter initial setting time.

### MTA Modification to Improve Setting Time

Another clinical problem is the setting time of the initial MTA formulation, which was reported to be greater than 3 h (Camilleri, 2015). When the material is utilized for root-end filling and repair of the perforation area, a long setting time is not a concern. When utilized as a PC or as a dressing over PP, however, faster setting time is required. A huge number of reports have surfaced involving the addition or substitution of various chemicals to the mixing liquid for water. The most common compounds are CaCl_2_, Ca(NO_3_)_2_/nitrate, and Ca(HOO)_2_. These additives are frequently used in the building industry to help Portland cement set faster. Setting accelerators affect both tricalcium silicate and tricalcium aluminate’s setting reactions. Mechanically mixed MTA uses the CaCl_2_ accelerator and has a faster setting time.

A study conducted by Saghiri et al., in 2020 on the evaluation of mechanical activation and chemical synthesis for particle size modification of WMTA showed that the use of the finer particle size in WMTA reduced the initial setting time that had previously been mentioned as a major drawback. When data from milling for 10 and 30 min was compared, 10 min was regarded as the optimal milling time for the powder (Saghiri et al., 2020). Further grinding resulted in a considerable increase in the surface area based on the Scanning Electron Microscope (SEM) particle size test. There was also a minor reduction in the setting time. The results showed that when compared to milling and control groups, the sol–gel approach produced finer powder with a smaller range of particles and normal powder particle distribution, resulting in higher compressive strength, pH, and calcium release rate of the powder (Saghiri et al., 2020). Both modification procedures had a considerable impact on powder quality, although the effect of the sol–gel process was significantly greater than milling, according to statistical analysis. The sol–gel method’s result was found to be finer, with a shorter distribution range and more homogeneous particles, than the other groups. As a result, both approaches investigated increased the surface area. Mechanical activation, on the other hand, was not as successful as the sol–gel technique in lowering the initial setting time (Saghiri et al., 2020). Furthermore, the sol–gel technique produced finer particles with a narrower size distribution range.

According to a study conducted by Kharouf et al., in 2021 titled “Tannic acid speeds up the setting of MTA cement and improves its surface and bulk properties.” Tannic acid (TA) can deposit on the grains’ surfaces (calcium oxide, calcium silicates, and trisilicates) and it can also be incorporated between the grains to change the porosity of the composite. The fact that the grains are coated with a polyphenol-based coating results in a much smaller pH increase when the cement is placed in water, which could also explain the cement’s faster setting kinetics. The presence of TA on the grain surface and in the pores explains the increase in compressive stiffness and stress in the dry state because TA molecules form strong intramolecular hydrogen bonds and hydrogen bonds/ionic interactions with the grain surface. As a result, adding TA to MTA cements reduces the setting time and grain size of the resulting cement while increasing the hydrophilicity of the composite materials (Kharouf et al., 2021).

### Modified MTA With Enhanced Compressive Strength and Microhardness

**FIGURE 4:**
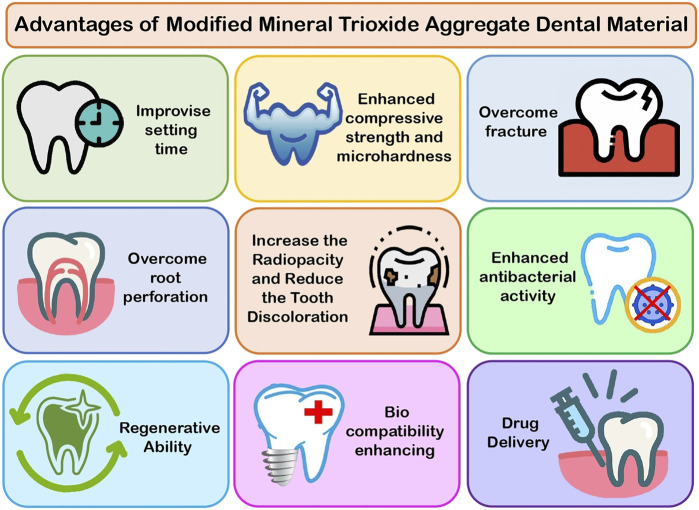
Advantages of modified MTA.

A study conducted by Eskandarinezhad et al., in 2021 on the effects of hydroxyapatite (HA) and zinc oxide (ZnO) nanoparticles on compressive strength of WMTA showed that the compressive strength of MTA was unaffected by HA or ZnO nanoparticles (Figure 4). As a result of the advantages of these nanoparticles, they can be used in cases where compressive strength is important, such as the repair of furcal perforations, pulp capping, and apexogenesis, or in cases where compressive strength is not important, such as the apical plug and as a retrograde material in surgery (Eskandarinezhad et al., 2020).

Kucukyildiz et al. conducted an *in-vitro* comparison of the physical, chemical, and mechanical properties of graphene nanoplatelet (GNP) with Angelus MTA. According to the Fourier transform infrared spectroscopy (FTIR) analysis, adding GNP to MTA did not change the binding structure of the material's atoms. According to energy dispersive X-ray spectroscopy (EDX) analysis, adding GNP to MTA did not change the crystal structure of the material but did result in an increase in the carbon ratio. By adding GNP to pulp capping materials to enhance hard tissue formation potential, the material’s microhardness can be increased, providing superior resilience under permanent restoration. The particle size of the MTA material is reduced, and its microhardness is increased as a result of adding GNP to the material; thus, GNP contributes to the MTA material’s strength. *In vivo* and *in vitro* research studies are needed to see how the GNP addition affects the biological effect on pulp (Kucukyildiz et al., 2021).

### Modification of MTA to Overcome Fracture

Endodontically treated teeth were long thought to be more susceptible to fracture than healthy teeth. This is because endodontic procedures can cause chemical and structural changes in the root canal dentin, making the tooth vulnerable to vertical root fracture (VRF). A variety of factors influence the brittleness of endodontically treated teeth. The use of root canal filler material to fortify the remaining tooth structure is one method of preventing VRF in endodontically treated teeth. Despite their poor adherence to the dentinal walls of the root canal system, gutta-percha and sealers have remained the standard of care for obturating root canals in endodontics (Fridland and Rosado, 2003). MTA has been proposed as having the ability to form a tighter seal with root dentin walls than many other available materials (Camilleri, 2015). Ballal et al. conducted a study to compare the fracture resistance of teeth obturated with two different types of MTA, ProRoot MTA and OrthoMTA and discovered that teeth obturated with OrthoMTA III had significantly higher fracture resistance than teeth obturated with ProRoot MTA. The difference in setting mechanisms could explain OrthoMTA III’s superior performance and pH neutralization during the reaction. As a result, enough H_2_O is produced to supply humidity to the dentin *via* dentinal tubules (Ballal et al., 2020).

The toughness of dentin is substantially higher in a hydrated state than in a dehydrated state. Another factor contributing to OrthoMTA III’s higher fracture resistance when compared to control and groups of ProRoot MTA is the interfacial adhesion between RC dentin walls and MTA. However, when compared to ProRoot MTA, the precipitate was more visible in the OrthoMTA III group. The toughness of dentin is substantially higher in a hydrated state than in a dehydrated state. Another factor contributing to OrthoMTA III’s higher fracture resistance when compared to control and groups of ProRoot MTA is the interfacial adhesion between RC dentin walls and MTA. Under current conditions, OrthoMTA III had a stronger biomineralization impact. Stronger interfacial bonding may be induced by this improved biomineralization impact. Within the dentinal tubules, petals-like precipitate are formed in the experimental groups. However, when compared to ProRoot MTA, the precipitate was more visible in the OrthoMTA III group. A study conducted by Żuk-Grajewska et al., in 2020 on the fracture resistance of MTA mixed with PBS in human roots filled with and without CaOH pre-medication showed that even when short-term calcium hydroxide pre-medication was utilized, MTA mixed with Ca and Mg free phosphate-buffered saline had a considerable strengthening impact on the fracture resistance of structurally weak roots. When MTA was mixed with water and pre-treated with calcium hydroxide for 2 or 12 weeks, the strengthening effect on human roots was lost (Żuk-Grajewska et al., 2021).

### Modification of MTA to Minimize Root Perforation

Root perforations are openings in the external root surfaces that allow communication between the root canal system and the root surface. It can be considered as pathogenic if it arises from defects in the resorptive area or caries, although the majority of them are because of iatrogenic causes during and after RC procedures. Perforations in the roots are one of the reasons for failure in endodontics, and if they are not detected and corrected promptly, they can result in the loss of the affected teeth. MTA has several desirable properties for endodontic repair, including strong sealing ability, biocompatibility, antibacterial properties, and hydrophilic behavior (Shin et al., 2021).

A study conducted by Adl et al., in 2019 on evaluation of the absence and presence of blood contamination, dislodgement resistance in a new pozzolan-containing calcium silicate-based substance when compared to ProRoot MTA and Biodentine showed that higher bond strength was seen in Biodentine and ProRoot MTA than in EndoSeal MTA (Adl et al., 2019). This result could be explained by the fact that pozzolan-based materials have a much lower releasing ability of Ca/P ratio and calcium of the apatite-like crystalline precipitate when compared to other calcium silicate cements. This viewpoint is supported by the fact that the concentration of accessible ions influences the nucleation and growth of the apatite layer, suggesting that they may be superior alternatives for furcal perforation repair (Shin et al., 2021). The final restoration should be placed within 7 days while using ProRoot MTA.

### MTA Modification to Improve Radiopacity and Reduce Tooth Discoloration

Bismuth oxide, the radiopacifying agent in MTA, becomes unstable when it interacts with strong oxidizing agents such as sodium hypochlorite or amino acids found in dentin collagen, resulting in tooth discoloration. Tooth discoloration occurs when this cement is used for additional treatment techniques such as vital pulp therapy, sealing root canal perforations, and root resorptions.

In a study conducted in 2020 by Bolhari et al. to evaluate the characteristics of different environmental conditions of MTA mixed with ZnO, it was discovered that the addition of 5% ZnO prevented tooth discoloration and decreased the compressive strength of both MTA Angelus and ProRoot MTA cement significantly in all exposure settings. Zinc hydroxide creates an impenetrable barrier around tricalcium silicate, preventing hydration of the cement. Because the composition and hydration of Portland cement and MTA are similar, this negative effect may occur in MTA as well, resulting in a reduction in compressive strength values. ZnO had no effect on the microhardness of these cements and was discovered on the surface of the cements to which it was added. Although adding 5% ZnO to MTA prevents tooth discoloration, it has a negative impact on the hydration and compressive strength of the tested cements but has no effect on the surface microhardness of Angelus and ProRoot MTA (Bolhari et al., 2020).

A study conducted by Peng et al., in 2021 on the spray pyrolysis of Zr-doped Bi_2_O_3_ radiopacifier on MTA showed bismuth oxide (Bi_2_O_3_) and zirconium (Zr)-doped Bi_2_O_3_ compounds were almost spherically produced. The particles had a tiny agglomeration on their surface, with the bigger particles (about 2 μm) having a small number of microscopic particles (0.5 μm) on their surface. Radiopacifiers made by spray pyrolysis had a faster setting time than those made by the sol–gel method. Furthermore, when compared to Bi_2_O_3_ and other ratios of Zr-doped Bi_2_O_3_ mixed with Portland cement, Bi_2_O_3_ with 15 mol% Zr doping had significantly better radiopacity and mechanical strength under X-ray excitation. The results support the use of Zr-doped Bi_2_O_3_ as a novel radiopacifier in future dental filling and pulp-capping applications *via* spray pyrolysis. Future research should concentrate on the effects of Zr in conjunction with Portland cement and the impact of tooth discoloration (Peng et al., 2021).

### Modification of MTA With Enhanced Antibacterial Activity

The success of endodontic treatment is intimately linked to the control of bacterial infection in the root canal. Various studies have linked five– seven bacterial species to root canal infection, with *Porphyromonas gingivalis*, *Porphyromonas endodontalis*, and *Enterococcus faecalis* being the most often isolated bacterial species from pulpitis. Apical periodontitis is linked to *Enterococcus faecalis* in RC-treated teeth, whereas *Porphyromonas endodontalis* and *Porphyromonas gingivalis* are related to the infection of the root canal (RC) (Shin et al., 2021). Because the root canal system is anatomically complex, the complete elimination of pathogenic microorganisms in the root canal is challenging.

A study conducted by Shin et al., in 2020 evaluated the antibacterial activity of a mixture of MTA and nitric oxide (NO)-releasing molecules against bacteria like *Enterococcus faecalis* and *Porphyromonas endodontalis* and the physical features of MTA and MTA-NO mixture to determine the efficacy of these techniques. When a NO-releasing molecule, such as diethylenetriamine-NO (DETA-NO), is mixed with powder of MTA, NO gets released quickly from DETA-NO before the mixture sets or during the initial phase, but the process slows down after the mixture sets (Shin et al., 2021). As a result, the high concentration of NO and mineral oxide and the mixture of MTA and NO had significant antibacterial action in the early stages and proved useful in eliminating oral microorganisms. Furthermore, bone formation and wound healing following endodontic treatment is due to NO released from the mixture at a low concentration.

### Modification of MTA With Regenerative Ability

Calcium silicate–based materials (CSBMs) have been used in a variety of endodontic procedures, including regenerative endodontics, root-end closure, vital pulp therapy, and perforation repair. These bioactive compounds have the ability to stimulate stem cell proliferation and differentiation into odontogenic/osteogenic cells. MTA, the first CSBM produced, was widely used in endodontic procedures. Angiogenesis is required for the mineralization of stem cells and the formation of extracellular matrix to recruit stem cells for oxygen and nutrient provision. Previous research has shown that angiogenic signaling molecules can aid stem cell proliferation and differentiation. Despite its high clinical success rate, a study discovered that MTA has decreased proangiogenic activity, which may influence the first cell-material contact and regeneration qualities (Almeshari et al., 2021).

To improve the angiogenic properties, a study was conducted by Almeshari et al., in 2021 to evaluate the additive effect of iloprost on the biological properties of MTA on mesenchymal stem cells. On day 7, MTA reduced cell viability. This result was explained by iloprost’s capacity to upregulate the proangiogenic factors and initiate proliferation of cells. In addition, MTA’s setting time, which is longer, may have also allowed for a more regulated and gradual release of iloprost. The cell morphological alterations on the material surfaces revealed human bone marrow-derived mesenchymal stem cells (hMSCs) had less spread of cells on the dentin disc, which was consistent with the assay of cell viability (Almeshari et al., 2021).The appearance of cells that were round on the material surface could be due to the leak of compounds, which influenced interaction between the cell and materials. On days 7 and 14, however, the substrate’s surface was covered with flat cells, demonstrating that the materials were biocompatible. hMSC migration throughout the first 24 h, resulting in a nearly 50% reduction in cell migration. When compared to MTA alone, iloprost had no effect on cell migration, which could be related to the grown cells due to the 2D environment. The results revealed that markers of osteogenic expression were dramatically elevated in MTA-iloprost-treated cells when compared to MTA-treated cells. This could be due to iloprost’s elevation of VEGF, which causes the production of osteogenic and odontogenic markers. Thus, adding iloprost to MTA increased the cell vitality and differentiation of osteogenic potential capability of mesenchymal stem cells, suggesting that it could be used as a biomaterial in the future.

A study conducted by Tien et al., in 2021 on additive manufacturing of caffeic acid-inspired MTA/poly-caprolactone scaffold for regulating vascular induction and osteogenic regeneration of dental pulp stem cells showed that the physicochemical and biological responses were caused by the surfaces of the various scaffolds that had been coated with caffeic acid (CA). The CA 20 scaffold not only had strong mechanical strength but also had apatite precipitate immersed in stimulated body fluid, indicating that this was the ideal physical and chemical microenvironment for human dental pulp stem cell (hDPSC) activities (Tien et al., 2021). Cell adhesion, proliferation, and osteogenic differentiation were all improved when the MTA scaffold was coated with a CA concentration of 20 mg/ml. Furthermore, VEGF adsorption resulted in an increase in CA coating on hDPSC osteogenesis differentiation. Most notably, *in vivo*, the MTA scaffold with CA covering demonstrated good bone regeneration. Furthermore, the CA 20 scaffold appeared to have better osteogenesis ability than the CA 0 scaffold, as determined by CT and histological analyses. These results could be related to the CA coating’s augmentation of bone-like apatite production and VEGF adsorption. Because CA is a bioinspired polymer, it can be used as a highly functional bioactive covering for various scaffolds in bone tissue creation and other biomedical applications. A study conducted by Ghasemi et al. (2021)on the effect of Ze–Ag–Zn on MTA on the odontogenic activity of hDPSCs showed that the incorporation of Ze–Ag–Zn particles into AMTA had no effect on the material’s biomineralization ability, and further research is needed to assess the underlying molecular interaction between HDPSCs and these types of particles, based on the findings of previous studies and their limitations.

A study conducted by Lim et al., in 2021 on *in vitro* evaluation of the mineralization and biocompatibility potential of MTA that included CaF_2_ showed that the addition of more than 5% calcium fluoride (CaF_2_) could be considered to increase pulp cell regeneration ability without compromising physical properties. However, clinical circumstances such as long-term irritation and inflammation following pulp contact may result in a different outcome (Lim et al., 2021).

Matrix metalloproteinases (MMPs), enzymes involved in bone remodeling, cells generated by osteoclasts, and other cells such as fibroblasts and macrophages, are all known to play a role in the restoration of the dental matrix. The main components of the extracellular matrix (ECM) act as metalloproteinases’ primary substrates. The MMP-induced catalysis causes structural changes in the matrix. MMPs have been found to perform a variety of roles, including regulating cell activity and participating in inflammatory reactions. MMPs have been shown to play an important regulatory role in cell differentiation and migration, growth factors, angiogenesis, and inflammation development.

### Modified MTA That Accelerates Biocompatibility

The introduction of tricalcium silicate materials that are resin modified has resulted in the development of a command cure material that may be used for PC. Resins improve the flow of material, making it acceptable for use as a sealer for RC. The liquid water mixing has been replaced by a variety of resins. As a result of these improvements, MTA that is light activated and MTA that is resin modified have been developed for the use in RC sealing cements. A variety of resin systems have been examined. The most common are light-curing systems, containing Bis-GMA and a resin that is biocompatible and contains HEMA, TEGDMA, camphorquinone, and EDMAB, with or without polyacrylic co-maleic acid, Bis-TEGDMA, Bis-GMA, PMDM, and HEMA (Camilleri, 2015).

TheraCal is a tricalcium silicate compound that is light curable and is used for PC. Environmental circumstances and fluid availability have an impact on TheraCal hydration. In reality, the restricted fluid availability limits material hydration when employed as a pulp-capping material. TheraCal’s ability to release calcium is controversial; in a study, it was shown to be comparable to CaOH, but later studies showed no calcium generated, and leaching of the calcium ion was found to be extremely low. When extracts of TheraCal came into contact with pulp cells, they were found to be cytotoxic. Angelus sells Fillapex of MTA is a modified MTA-based resin. MTA is a salicylate resin, and additional ingredients make up this mixture (Camilleri, 2015).

The inclusion of polymers that are soluble in water showed that in prototype materials it can improve material flow. ProRoot Endo Sealer is a commercially available product that contains particles that make up the cement suspended in a polymer that is soluble in water. At low water/cement ratios, the polymer that is soluble in water puts the cement particles on charge. The resultant charged particles repel each other, resulting in less flocculation and enhanced flow of material. Another soluble polymer, propylene glycol, has been utilized to increase MTA flow.

In a study conducted by Toida et al., in 2021 on pulp response to capping material flow that is phosphorylated pullulan-based in MTA (MTAPPL), it was shown that the group had reduced inflammatory cell infiltration, whereas the Nex-Cem MTA (NX) and Dycal (DY) groups had more inflammatory cells with infiltration of polymorphonuclear leucocytes at the exposed site of pulp (Kim et al., 2011). According to the findings, MTAPPL had lower inflammatory responses than the other experimental groups at all time points. The study also revealed that with the exception of the DY group, the deposition of tissue that was mineralized was seen in the MTAPPL, NX, and TheraCal LC (TH) groups. This finding can improve the sealing ability, inhibit bacterial leakage, and boost the reparative capacity of pulp cells by depositing calcium phosphate minerals along the dentin-material contact. After 70 days, the MTAPPL, TH, and NX groups showed more mineralized tissue formation than the DY group. Tubular dentin grew under the osteodentin and was also identified as layers of odontoblast that were well arranged with odontoblast-like cells. There were no tunnel defects in any groups. However, some pulp-like tissue was found at the tissue barrier that was mineralized in the MTAPPL group. This could be due to the faster formation of mineralized tissue. MTAPPL’s ability to adhere to human dentin was compared to NX, TH, and DY. There was no separation between MTAPPL and dentin in the experiment, even under the high-vacuum conditions of the SEM sample chamber, indicating that it has outstanding sealing ability as a pulp-capping material. On the other hand, NX, TH, and DY did not stick well to dentin and demonstrated separation from dentin interface. Leakage and bacterial infection may occur as a result of these holes. As a result, MTAPPL biomaterials have good dentin sealing ability and pulpal responsiveness (Toida et al., 2021). MMP-2 and MMP-9 protein expressions in cultured monocytes/macrophages are unaffected by MTA Repair HP, according to a study conducted by Barczak et al. (2021) to investigate the MTA Repair HP formula’s effect on the inflammation process, involving the tooth and periodontal tissues using the THP-1 monocyte/macrophage model with biomaterial applied in direct contact with the cells.A study conducted by Tabari et al., in 2020 on an animal study on the biocompatibility of MTA mixed with different accelerators showed the inflammatory response diminished over time for all of the accelerators, that is, sodium hypochlorite (Na_2_HPo_4_), citric acid, and calcium lactate gluconate (CLG) studied in this investigation. The inflammatory responses caused by MTA combined with 0.1 percent citric acid and MTA mixed with 15 percent Na_2_HP0_4_ were equivalent to those induced by MTA mixed with pure water, according to the findings of this investigation. Despite the lack of substantial differences in histological reactions between control MTA and MTA mixed with CLG, the latter cement elicited a moderate-to-severe inflammatory response on the 7th day after implantation (Tabari et al., 2020).

In another study, pulpal response to the combined use of mineral trioxide aggregate and iloprost for direct pulp capping, conducted by Adl et al., in 2021 found that when used as pulp-capping materials, iloprost, MTA, and MTA-iloprost all produced positive results. All materials demonstrated biocompatibility and the formation of hard tissue. Because it increases the mRNA expression of VEGF, fibroblast growth factor-2, and platelet-derived growth factor in human dental pulp stem cells (Adl et al., 2019). As a result, the combination of MTA and iloprost may have a little therapeutic value. A study conducted by Kim et al., in 2020 on the investigation of properties of an unique experimental elastin-like polypeptide-based MTA as an endodontic sealer showed that the experimental elastin-like polypeptide (ELP)-based MTA sealer performed better in binding strength to dentin and flow rate than the other experimental groups at a 0.4 L/P ratio (Kim et al., 2021). In addition, when compared to commercial MTA sealers, it has the same or better quality in terms of dentinal tubule penetration and outstanding washout resistance. As a result, if additional research and clinical trials confirm its qualities, the ELP-based MTA sealer may be approved for clinical use. Incorporating V125E8 a 125 times-repeated structure of a valine–proline–glycine–valine–glycine unit with eight glutamates added on the C-terminus into MTA could be a promising way to develop functionally advanced bioactive sealers for endodontic therapy and regenerative endodontics.

### Modified MTA as a Drug Delivery

MM-MTA is manufactured by Micro-Mega (Besancon, Cedex, France), and it uses MTA capsules that can be blended using an amalgamator. The MM-MTA delivery system has the advantage of including a delivery method comparable to glass ionomer cements. The compressive strength of the set material was proven to be increased, but the efficiency of mechanical mixing remains unknown. Another study found that mixing different processes had no significant effect on the resulting MTA mixtures, despite increased material microhardness (Camilleri, 2015). Incorporation of amoxicillin-loaded microspheres in MTA cement: an *in vitro* study, conducted by Bohns et al., in 2020, demonstrated the development of bioactive cement using polymeric microspheres to provide antibiotics with delayed release as a viable drug delivery mechanism for treating local tooth infections. The synthesis result is simple, repeatable, and extremely reliable. The use of polymeric microspheres at a concentration of 5% results in delayed release with a minimal impact on the physical and mechanical properties of the material. Limiting the release of antibiotics administered at local infection sites could have applications in dental materials that come into close contact with tooth tissues (Bohns et al., 2020). Table 2 summarizes the changes made to the MTA to address flaws of conventional MTA.

**TABLE 2:** Summary of studies relevant to modifications of MTA.

Author and year	Agents incorporated	Properties modified	Result
Saghiri et al. (2020), Duarte et al. (2005)	Particle size modification of WMTA using the sol–gel method	Setting time	• Reduction in the setting time
			• Higher compressive strength
			• Increased the surface area
Kharouf et al. (2021), Salem-Milani et al. (2017)	Incorporation of tannic acid	Setting time	• Strong intramolecular hydrogen bonds
			• Increase in compressive stiffness and peak compressive stress in the dry state
			• Reduces the setting time and grain size
			• Increases composite materials’ hydrophilicity
Eskandarinezhad et al. (2021), Kogan et al. (2006)	Hydroxyapatite (HA) and ZnO nanoparticles in WMTA	Compressive strength	• Compressive strength of MTA was unaffected by HA or ZnO nanoparticles
Kucukyildiz et al. (2020), Camilleri et al. (2005)	Graphene nanoplatelet in Angelus MTA	Microhardness	• No modification in the binding structure of the material’s atoms observed
			• No change in crystal structure noticed
			• Increase in microhardness and strength
			• Superior resilience under permanent restoration
Ballal et al. (2020), Fridland and Rosado, (2003)	Ortho MTA III	Fracture resistance	• Higher fracture resistance
			• Superior performance and neutralization of elevated pH during the reaction
			• Stronger biomineralization
			• Higher compressive and flexural strengths than ProRoot MTA
Żuk-Grajewska et al. (2020), Coomaraswamy et al. (2007)	MTA mixed with PBS in human roots filled with and without CaOH pre-medication	Fracture resistance	• MTA mixed with Ca and Mg free phosphate-buffered saline had a strengthening impact on the fracture resistance
Adl et al. (2019), Parirokh and Torabinejad, (2010b)	New pozzolan-containing calcium silicate–based material	Dislodgement resistance	• Higher bond strength
Bolhari et al. (2020), Asgary et al. (2005)	MTA mixed with different concentration of ZnO	Tooth discoloration	• 5% ZnO prevented tooth from discoloration
			• Reduction in compressive strength
			• Negative impacts on the hydration
Peng et al. (2021), Torabinejad et al. (1995)	Spray pyrolysis of zirconium-doped bismuth oxide radiopacifier	Radiopacity	• Faster setting time
			• Bi₂O₃ with 15 mol% Zr doping exhibited significantly better radiopacity
			• Increased mechanical strength
Shin et al. (2021), Lee, (2000)	Mixture of MTA and NO-releasing molecule	Antibacterial activity	• Significant antibacterial action in the early stages and useful in eliminating oral microorganisms
			• Bone formation and wound healing property
Almeshari et al. (2021), Boutsioukis et al. (2008)	Additive effect of iloprost with MTA	Regenerative ability	• Capacity to upregulate the proangiogenic factors and initiate proliferation of cell
			• Biocompatible
			• Marker of osteogenic expression dramatically elevated in MTA-iloprost-treated cells
			• MTA increased the cell vitality and differentiation of osteogenic potential capability
Tien et al. (2021), Mathew et al. (2021)	Caffeic acid-inspired MTA	Regenerative ability	• Strong mechanical strength
			• Ideal physical and chemical microenvironment for hDPSC activities
			• Cell adhesion, proliferation, and osteogenic differentiation improved
			• Improved osteogenesis differentiation
			• Good bone regeneration
Ghasemi et al. (2021), Formosa et al. (2013)	Ze–Ag–Zn to MTA	Regenerative ability	• No effect on the material’s biomineralization
			• Further research is needed to assess the underlying molecular interaction
Lim et al. (2021), Kim et al. (2011)	Addition of calcium fluoride to MTA	Mineralization	• Increase pulp cell regeneration
Toida et al. (2021), Ha et al. (2016)	Addition of phosphorylated pullulan and TheraCal in MTA	Biocompatibility	• Reduced inflammatory cell infiltration
			• Deposition of tissue that was mineralized was seen
			• Improve the sealing ability, inhibit bacterial leakage, and boost the reparative capacity of pulp cells
			• Mineralized tissue formation
			• Layers of odontoblast seen
Barczak et al. (2021), Saghiri et al. (2020)	MTA Repair HP with application of the THP-1 monocyte/macrophage model	Biocompatibility	• Protein expression in cultured monocytes/macrophages is unaffected by MTA Repair HP
Tabari et al. (2020), Kharouf et al. (2021)	MTA mixed with different accelerators such as sodium hypochlorite, citric acid, calcium lactate gluconate	Biocompatibility	• Inflammatory responses of sodium hypochlorite and citric acid was similar to conventional MTA
			• Calcium Lactate Gluconate elicited moderate-to-severe inflammatory response
Almeshari et al. (2021), Boutsioukis et al. (2008)	MTA with iloprost	Biocompatibility	• Showed biocompatibility and the development of hard tissue
			• It upregulates the mRNA expression of vascular endothelial growth factor, fibroblast growth factor-2, and platelet-derived growth factor in human dental pulp stem cells
Kim et al. (2021), Eskandarinezhad et al. (2020)	MTA with elastin-like polypeptide	Biocompatibility	• Better in binding strength to dentin and flow rate
			• Same or better quality of sealer in terms of dentinal tubule penetration and washout resistance
Bohns et al. (2020), Kucukyildiz et al. (2021)	Incorporation of amoxicillin-loaded microspheres in MTA	Drug delivery	• Delayed release with minimal impact on the material’s physical and mechanical properties
Almeida et al. (2018), Ballal et al. (2020)	MTA-based endodontic sealer with calcium aluminate (C3A) and silver-containing C3A particles	Antibiofilm	• Antibiofilm effect was improved in the presence of C3A particles, while the biofilm inhibition was lower in the presence of Ag
			• Physicochemical properties of the modified MTA-based sealer were similar to the commercial material
			• Significant increase in Ca^+2^ release
Hernandez-Delgadillo et al. (2017), Żuk-Grajewska et al. (2021)	MTA mixed with bismuth lipophilic nanoparticles (BisBAL NPs)	Antibiofilm and antimicrobial	• MTA-BisBAL NPs inhibited the growth of *Enterococcus faecalis, Escherichia coli*, and *Candida albicans*, and also detached the biofilm of fluorescent *Enterococcus faecalis* after 24 h of treatment
			• Cytotoxicity was not observed when MTA-BisBAL NPs was added on human gingival fibroblasts
Nagas et al. (2016), Shin et al. (2021)	Mixing alkaline resistant(AR) glass fibers in ProRoot MTA	Fracture resistance	• Highest fracture strength
			• Higher diametral tensile strength and compressive strength
Marciano et al. (2019), Adl et al. (2019)	Mixing MTA Angelus with aluminum fluoride	Tooth discoloration	• Did not significantly alter the radiopacity, setting time, and volume change
			• pH and calcium ion release significantly increased
			• Prevented discoloration
			• Did not interfere in inflammatory response
Siboni et al. (2017), Bolhari et al. (2020)	Incorporation of povidine and polycarbonate	Radiopacity	• Had bioactivity with calcium release
			• Strong alkalizing activity and apatite-forming ability
			• Adequate radiopacity
Dianat et al. (2017), Peng et al. (2021)	MTA with methyl cellulose as liquid	Mechanical properties	• Using methyl cellulose as the hydrating liquid enhance some mechanical properties but does not compromise pH of white ProRoot MTA

## Conclusion

MTA’s introduction was recognized as a watershed moment in material science, and its properties have since been improved to maximize its benefits. It is the most widely used endodontic material by dentists because it has been demonstrated to be effective in a variety of clinical settings. However, this substance has always had a few limitations that have prompted researchers all over the world to look for alternatives. The majority of newer formulations contain additives that improve material properties. With the recent introduction of new, improved MTA products, novel tricalcium silicate-based materials have surpassed MTA’s major applications. MTA-based materials are still widely used due to their outstanding properties and regeneration capacity. Although more long-term research is needed to confirm this concept, the novel materials could still be viewed as a possible alternative to MTA.
